# Molecular Mechanisms of Resistance to Bispecific Antibodies in Diffuse Large B-Cell Lymphoma

**DOI:** 10.3390/cells15090794

**Published:** 2026-04-27

**Authors:** Nawar Maher, Bashar Al Deeban, Ndeye Marie Diop, Joelle Assaf, Riccardo Moia, Samir Mouhssine, Gianluca Gaidano

**Affiliations:** Division of Hematology, Department of Translational Medicine, Università del Piemonte Orientale, Alessandria and Novara, Italy; nawar.maher@uniupo.it (N.M.);

**Keywords:** diffuse large B cell lymphoma, immunotherapy, bispecific antibody, drug resistance, microenvironment

## Abstract

CD20 × CD3 bispecific antibodies (BsAbs) have emerged as a meaningful therapeutic option for relapsed or refractory diffuse large B-cell lymphoma (DLBCL), redirecting endogenous T cells against malignant B cells independently of major histocompatibility complex-mediated antigen presentation, and have received regulatory approval after at least two prior lines of therapy. However, a substantial proportion of patients experience primary resistance or early relapse, underscoring the need to characterize the underlying biological mechanisms, which are the focus of this review. Several tumor-intrinsic determinants of resistance have been identified, including CD20 loss driven by *MS4A1* mutations, alternative splicing, and gene deletion, as well as genomic reprogramming involving *TP53*, *MYC*, and *NOTCH1* alterations. T-cell dysfunction represents another critical resistance domain, encompassing inadequate intratumoral cytotoxic CD8+ T-cell infiltration, expansion of immunosuppressive regulatory and follicular helper T cells, progressive exhaustion with upregulation of PD-1, LAG-3, TIM-3, and TIGIT, and impaired T-cell fitness from prior treatment exposure. Microenvironmental barriers, including checkpoint ligand upregulation, PD-L1-enriched extracellular vesicles, spatial exclusion of effector cells from immune-cold germinal center-like niches, hypoxia, and metabolic competition, further reinforce immune escape. Emerging strategies to overcome resistance include epigenetic priming, checkpoint inhibitor combinations, 4-1BB costimulatory approaches, and next-generation multispecific antibody designs.

## 1. Introduction

Diffuse large B-cell lymphoma (DLBCL) is the most common subtype of non-Hodgkin lymphoma (NHL) and represents an aggressive malignancy characterized by substantial biological and clinical heterogeneity [1,2,3]. Frontline chemoimmunotherapy with rituximab combined with cyclophosphamide, doxorubicin, vincristine and prednisone (R-CHOP) has remained the standard of care for more than two decades and achieves long-term remission in approximately 60–65% of patients. More recently, the incorporation of the antibody–drug conjugate polatuzumab vedotin into frontline therapy (Pola-R-CHP) has demonstrated improved progression-free survival in patients with previously untreated DLBCL and represents an important advancement in first-line treatment [4]. Despite these advances, approximately 30–40% of patients develop relapsed or refractory (R/R) disease, which is associated with poor outcomes and represents a major unmet clinical need [5].

Historically, salvage chemotherapy followed by autologous stem cell transplantation (ASCT) has been the preferred approach for transplant-eligible patients with chemosensitive relapse [6]. However, many patients are not eligible for ASCT because of age, comorbidities, or refractory disease, and long-term remission is achieved in only a subset of patients [7]. More recently, the development of novel immunotherapies, including antibody–drug conjugates and chimeric antigen receptor (CAR) T-cell therapy, has expanded treatment options for R/R DLBCL [8,9,10]. CAR T-cell therapy, in particular, has demonstrated durable responses in heavily pretreated patients, although its use remains limited by manufacturing complexity, treatment delays, toxicity, and accessibility [11,12]. These limitations have stimulated interest in alternative strategies that redirect endogenous immune cells against lymphoma cells.

Bispecific antibodies (BsAbs) have emerged as a novel immunotherapeutic approach capable of redirecting T cells toward malignant B cells [13]. These engineered antibodies simultaneously bind CD3 on T cells and a tumor-associated antigen, most commonly CD20, on lymphoma cells, creating an artificial immune synapse that activates T-cell cytotoxicity [14]. Importantly, this process occurs independently of major histocompatibility complex (MHC)-mediated antigen presentation, allowing effective tumor cell killing even in lymphomas with impaired antigen presentation [15]. Following engagement with tumor cells, activated T cells release cytotoxic granules and inflammatory cytokines that promote targeted tumor cell apoptosis and the recruitment of additional immune effector cells. Several CD20 × CD3 BsAbs, including epcoritamab, glofitamab, odronextamab, and mosunetuzumab, have demonstrated encouraging activity in clinical trials involving heavily pretreated patients with DLBCL [16,17,18,19,20]. Based on these results, epcoritamab and glofitamab have been approved for patients with R/R DLBCL after at least two prior lines of therapy [21,22,23].

Compared with cellular therapies, BsAbs offer several practical advantages, including off-the-shelf availability, rapid treatment initiation, and the ability to administer repeated dosing [24]. These features make CD20 × CD3 T-cell engagers (TCEs) an attractive therapeutic strategy in aggressive B-cell lymphomas. However, despite their promising clinical activity, a significant proportion of patients fail to achieve durable responses, underscoring the need to better understand the biological mechanisms underlying resistance to these agents [25]. In this review, we summarize the current knowledge regarding the mechanisms of resistance to CD20 × CD3 BsAbs in DLBCL, focusing on tumor-intrinsic factors, T-cell dysfunction, and microenvironmental influences that may limit the efficacy of these therapies and inform the development of future therapeutic strategies.

## 2. Tumor Cell Determinants of Resistance

**Figure 1:**
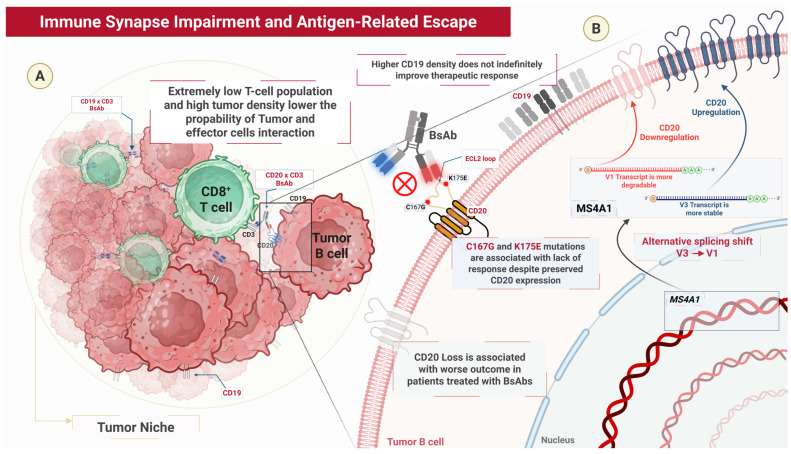
Genetic determinants of resistance to T cell engagers therapy. (**A**) The spatial distribution of cells within the tumor niche influences therapeutic efficacy. Low T-cell infiltration relative to high tumor burden reduces the probability of effective interactions between CD8^+^ T cells and malignant B cells. Although bispecific antibodies (BsAbs; CD19 × CD3, CD20 × CD3) bridge CD3 on T cells to CD19 or CD20 on B cells, an unfavorable effector-to-target ratio impairs immune synapse formation and limits cytotoxic activity. (**B**) Tumor-intrinsic mechanisms drive antigen escape. CD20 loss is associated with resistance and poor clinical outcomes. Mutations in the extracellular loop 2 (ECL2) of CD20 (namely, C167G, K175E) disrupt BsAb binding while preserving surface expression. In addition, alternative splicing of *MS4A1* from the stable V3 to the more degradable V1 transcript reduces CD20 expression, further limiting target availability.

Resistance to BsAbs can arise from several tumor cell-intrinsic factors that impair effective immune-mediated killing [26]. Among these, defects in immune synapse formation and antigen-related escape play an important role, as reduced antigen expression or loss of the target molecule can limit T-cell engagement and cytotoxic activity [27]. In addition, tumor cells may acquire resistance through genetic reprogramming [28]. This can occur through alterations affecting key regulatory pathways, mainly due to onco-suppressor gene disruption (such as *TP53* loss-of-function mutations or deletions) or oncogene-activating mutations (such as *MYC* and *NOTCH1* gain-of-function mutations) [28,29]. Together, these tumor-intrinsic mechanisms contribute to disease persistence and progression during BsAb therapy (Figure 1).

### 2.1. Immune Synapse Impairment and Antigen-Related Escape

Immune synapse formation during BsAbs therapy is driven by two fundamental processes: the encounter between T cells and tumor cells and their subsequent adhesion [30]. The likelihood of these encounters largely depends on the density and spatial distribution of both cell populations. B-cell lymphoma *in vitro* and *in vivo* models suggest that when T-cell numbers are low, such as in the bone marrow, tumor cells are less likely to interact with effector cells, allowing them to survive longer and potentially develop mechanisms of immune evasion to the CD19 × CD3 BsAb blinatumomab [27]. Conversely, extremely high tumor cell densities can also reduce treatment efficacy because the available antibody may be insufficient to mediate effective synapse formation across all targets.

Once cells meet, adhesion is influenced by molecular factors, including BsAbs concentration, binding affinity, and antigen expression such as CD19. While increasing CD19 expression enhances tumor cell killing when levels are low, this effect reaches its plateau beyond a certain threshold, indicating that higher antigen density does not indefinitely improve therapeutic response. In contrast, tumor cell density has a dual effect: moderate increases improve encounter probability, whereas excessive densities limit antibody-mediated interactions [27].

Additionally, mechanisms of immune escape similar to those observed with CD19-directed CAR T-cell therapy have been described, with antigen loss representing one of the most frequent [28,31,32]. Grigg et al. evaluated 41 patients with R/R DLBCL or primary mediastinal B-cell lymphoma (PMBCL) treated with glofitamab after a median of three previous lines of therapy [33]. At relapse, CD20 loss was observed in 59% of cases, and patients had a median overall survival (OS) of 4.1 months after progression. Similarly, CD20 expression was assessed in tumor samples from patients with R/R B-NHL treated with mosunetuzumab monotherapy using immunohistochemistry, RNA sequencing, and whole-exome sequencing (WES) [34]. Before treatment, 10.9% of patients showed intermediate CD20 expression levels (10–74%), while a smaller subset (5.5%) exhibited very low expression (<10%). These reduced levels were associated with poorer clinical outcomes, suggesting that baseline assessment of CD20 expression may have prognostic relevance [34]. WES identified fourteen CD20 variants arising from mutations located either in the extracellular loop or in the transmembrane domain; however, variants in the transmembrane region were not associated with reduced CD20 expression. In contrast, two missense mutations (C167G and K175E) affecting extracellular loop 2 (ECL2) of the *MS4A1* gene, which contains the binding site for therapeutic anti-CD20 antibodies, were associated with a lack of response despite preserved CD20 expression [34].

Further evidence supporting the role of *MS4A1* alterations was provided by an analysis of missense mutations affecting the gene and reported an association between rapid DLBCL relapse and reduced CD20 expression and stability caused by these genetic alterations following rituximab exposure [35]. These findings suggest that prior anti-CD20 therapies may influence the effectiveness of CD20 × CD3 BsAbs. Additional mechanisms contributing to CD20 loss involve alternative splicing [36]. After identifying four CD20 transcript variants (V1–V4) characterized by distinct 5′untranslated regions (UTRs), Ang et al. demonstrated that splicing alterations can drive resistance to mosunetuzumab [36]. In particular, a shift from the V3 to the V1 transcript variant resulted in CD20 downregulation in relapsed lymphoma, indicating that alternative splicing may contribute to CD20-negative relapses. CD20 loss has also been associated with the deletion of *MS4A1*, which correlates with poor clinical outcomes [37,38]. Consistently, with a median follow-up of 18.3 months after anti-CD20 therapy, an analysis reported that CD20-negative R/R follicular lymphoma was associated with significantly shorter OS (8.9 months; 95% CI 2.4–19.1) compared with CD20-positive disease (28.3 months; 95% CI 25.1–75.3) [38]. In addition, Duell et al. used WES to show that truncating mutations of *MS4A1* were present in approximately 80% of patients who progressed after CD20 × CD3 BsAb therapy [39].

Despite these findings, the role of CD20 loss in resistance remains incompletely understood, as clinical responses to currently approved BsAbs have also been reported in patients with progressive disease [40]. Supporting this observation, Schuster et al. analyzed 293 patients with R/R B-NHL treated with mosunetuzumab administered in three-week cycles and found that CD20 expression was preserved in most patients who subsequently experienced disease progression [34,41]. These results suggest that additional resistance mechanisms, beyond antigen loss, may contribute to relapse, similarly to what has been described for CD19-positive relapses following CAR T-cell therapy [28].

### 2.2. Genetic Reprogramming

Beyond antigen loss, several genomic alterations have been implicated in the development of resistance to BsAbs in B-cell lymphomas. Resistance to glofitamab has been associated with transcriptional changes characterized by the downregulation of p53-dependent pathways and the upregulation of MYC-related targets. Consistently, an increased frequency of *TP53* mutations has been reported in patients with DLBCL who experience disease progression [31,42].

Kyvsgaard et al. investigated pretreatment genomic alterations in 56 patients with B-NHL treated with CD20 × CD3 BsAbs between 2017 and 2023 using next-generation sequencing (NGS) with a customized lymphoma gene panel [43]. Alterations in *NOTCH1* were associated with reduced survival, and the expansion of abnormal clones carrying these mutations was observed during therapy, suggesting a potential role in the development of BsAbs resistance. Mechanistically, activating *NOTCH1* mutations (N1 subtype) drive resistance by triggering persistent NICD release and upregulating *HES1*/*HEY1*, which directly inhibit CD8^+^ T-cell function [44]. Furthermore, recent evidence suggests that aberrant NOTCH activation can also be triggered by *CREBBP*/*EP300* mutations via the FBXW7-NOTCH-CCL2/CSF1 axis, which orchestrates the recruitment and M2 polarization of TAMs [45]. This coordinated reprogramming creates a metabolic and immunological barrier that prevents redirected T cells from effectively eliminating tumor cells despite successful BsAb engagement.

Also, MYC upregulation acts as a functional driver of BsAb resistance by orchestrating a metabolic-immune suppressive axis within the TME [26,44]. It drives BCR extinction, which allows tumor cells to uncouple transcriptional output from mTOR-mediated translation to facilitate survival under therapeutic stress [46]. Also, high MYC signatures are a key component of the “Immune Exhaustion-Related Prognostic Score” (IERPS), which characterizes a state of profound endogenous T-cell failure. This “immune-exhausted” phenotype is marked by a striking expansion of PD-1^+^ CD8^+^ T cells and the depletion of PD-L1^+^ B cells, representing a differentiation endpoint of terminal dysfunction that tracks poor longitudinal responses to glofitamab [47].

Further insights were provided by an analysis of circulating tumor DNA (ctDNA) from 41 patients with R/R large B-cell lymphoma (LBCL) treated with glofitamab using CAPP-seq. Mutations were frequently detected in genes including *TP53* (44%), *KMT2D*, *PIM1*, and *IGLL5* (37% each), *CARD11* (27%), *HIST1H1E* and *CREBBP* (24% each), and *BCL2* (22%) [48]. Although *TP53* mutations, present in 44% of cases (18/41 patients) before starting glofitamab, were not directly associated with worse PFS, their persistence after the third cycle of therapy was predictive of subsequent disease progression, highlighting the potential value of incorporating longitudinal ctDNA data into dynamic models to better capture resistance evolution over time. p53 loss triggers a suppressive microenvironment via the secretion of Interleukin-34 (IL-34) by cancer stem cells. This cytokine orchestrates the metabolic reprogramming of TAMs through the CD36 axis into an M2-like “foam” phenotype, which creates an immunosuppressive niche that actively excludes CD8+ T cells and suppresses their cytotoxic potential [49]. This “immune-cold” state is further reinforced by the upregulation of PD-L1 and the downregulation of MHC-II following p53 loss, which exacerbates T-cell exhaustion and increases the expression of inhibitory markers like LAG-3 and TIM-3 [44].

Overall, these findings highlight the complexity of gene reprogramming in the context of resistance mechanisms to BsAbs in B-cell lymphomas. Continued investigation of the genetic and epigenetic landscape of these malignancies will be essential to identify additional drivers of therapeutic resistance and of clonal evolution in order to guide the development of more effective treatment strategies.

## 3. T-Cell Dysfunction

**Figure 2:**
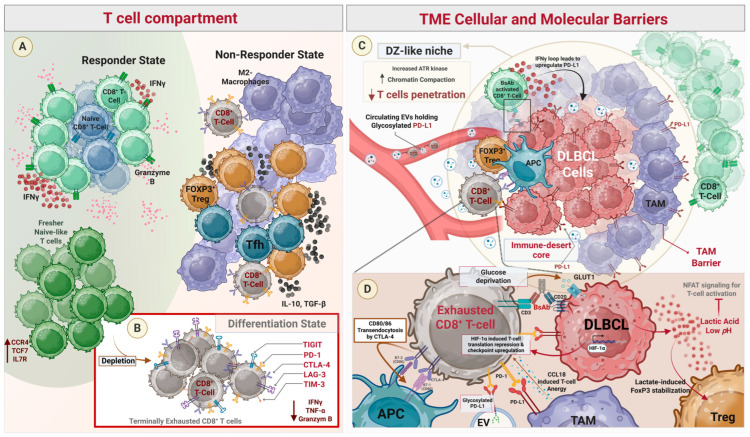
Immune dysfunction driving resistance to BsAbs in DLBCL. (**A**) Responders to BsAbs exhibit increased cytotoxic CD8^+^ T cells and naïve-like T cells expressing CCR7, TCF7, and IL7R, whereas non-responders show depletion of these populations and enrichment of immunosuppressive cells, including M2 macrophages, FOXP3^+^ regulatory T cells, and T-follicular helper cells, along with elevated IL-10 and TGF-β. (**B**) Resistance to BsAbs is associated with T-cell terminal exhaustion, characterized by upregulation of inhibitory receptors (PD-1, CTLA-4, TIGIT, LAG-3, TIM-3) and reduced effector function (IFN-γ, granzyme B). (**C**) The tumor microenvironment (TME) limits BsAb activity through spatial and molecular barriers, including DZ-like niches with ATR-driven chromatin compaction preventing T-cell penetration, T-cell exclusion from tumor cores, and macrophage barriers. IFN-γ-induced PD-L1 expression and PD-L1-bearing extracellular vesicles further suppress immunity. (**D**) Metabolic and molecular mechanisms impair T-cell function, including CTLA-4-mediated transendocytosis of CD80/86, tumor-driven glucose competition via GLUT1, HIF-1α-associated T-cell translational suppression, lactic acidosis-induced FoxP3 stabilization in Treg, and TAM-derived CCL18 promoting T-cell anergy.

T-cell-redirecting BsAbs rely on the presence of functional T cells capable of recognizing and eliminating tumor cells after CD3 engagement [50]. Consequently, the composition, differentiation state, and functional fitness of the T-cell compartment, within both the tumor microenvironment and peripheral circulation, critically influence therapeutic efficacy. Increasing evidence indicates that alterations in T-cell subsets, immune contexture, and activation–exhaustion dynamics can shape responses to CD20/CD3 BsAbs and contribute to treatment resistance in DLBCL (Figure 2) [28].

### 3.1. T-Cell Composition and Intratumoral Immune Landscape

The composition of the intratumoral T-cell compartment also plays a key role in determining the efficacy of T-cell redirection [51]. Single-cell immune profiling of lymphoma biopsies prior to BsAbs treatment has shown that clinical responses are associated with the clonal expansion of cytotoxic CD8^+^ T cells and cytotoxic CD4^+^ T-cell populations within the tumor microenvironment [52]. These effector populations exhibit transcriptional programs characterized by the expression of cytotoxic molecules such as granzyme family members, perforin, and interferon-stimulated genes. In contrast, tumors from patients with progressive disease frequently demonstrate depletion of cytotoxic CD8^+^ T cells and enrichment of non-cytotoxic CD4^+^ populations, including follicular helper T cells (Tfh) and regulatory T cells (Tregs) [52]. Functional experiments further confirm that BsAbs-mediated tumor killing is primarily driven by CD8^+^ T cells, whereas Tfh and Treg populations possess limited cytotoxic potential and may compete for CD3 engagement, thereby reducing effective tumor cell killing.

Tregs, particularly those expressing CD4 and FOXP3, are key mediators of immune evasion in the DLBCL tumor microenvironment [53,54]. In longitudinal studies of patients treated with glofitamab, those who developed drug resistance showed a significant increase in the proportion of CD4^+^ Treg cells within the primary tumor microenvironment [55]. Similarly, patients experiencing disease progression on epcoritamab demonstrated a clonal expansion of Treg and Tfh cells, whereas responders showed an expansion of cytotoxic T cells [50]. These cells suppress the activity of cytotoxic T lymphocytes by releasing inhibitory cytokines such as IL-10 and TGF-β and by expressing checkpoint molecules like PD-L1 [53].

In addition, resistance to CD20/CD3 BsAbs therapies has been associated with a marked transition of CD8^+^ effector T cells into CD8^+^ exhausted T cells. Consistent with these findings, alterations in specific T-cell subpopulations have been observed during glofitamab treatment, particularly among CD4^+^ Treg cells, CD8^+^ effector T (Teff) cells, CD8^+^ exhausted T cells, and two subsets of CD8^+^ naïve T cells. Single-cell TCR sequencing further demonstrated a transition from CD8^+^ effector T cells to CD8^+^ exhausted T cells [56].

### 3.2. T-Cell Differentiation State and Functional Fitness

Beyond T-cell abundance, the functional state of the T-cell compartment has emerged as a key determinant of therapeutic response [50]. Longitudinal single-cell transcriptomic analyses of peripheral blood immune cells from patients treated with glofitamab have revealed distinct T-cell states associated with clinical outcomes. In particular, patients achieving complete metabolic responses demonstrate maintenance of naïve-like or “fresher” T-cell states during early treatment, whereas non-responders exhibit a progressive decline in these populations over time [56]. These naïve-like signatures are defined by the expression of genes such as CCR7, TCF7, IL7R, and SELL and are associated with greater proliferative capacity and functional plasticity. Notably, the naïve-like signature observed in responders appears to be enriched within cytotoxic effector populations, suggesting the presence of “fresher cytotoxic T cells” that retain both effector function and stem-like proliferative potential [56]. In contrast, non-responders demonstrate reduced representation of these populations [56]. Importantly, treatment with glofitamab induces activation of both cytotoxic and exhaustion programs within T cells in responders and non-responders alike. Transcriptional analyses demonstrate increased cytotoxicity and exhaustion scores during therapy, reflecting ongoing immune activation triggered by CD3 engagement. However, responders maintain a higher proportion of naïve-like T cells, whereas non-responders display a more pronounced shift toward exhausted states, particularly during later treatment cycles [56]. This observation suggests that the preservation of less-differentiated T-cell states may allow sustained immune activation while preventing terminal dysfunction.

Functional assays further support the importance of T-cell fitness in determining therapeutic outcomes. Ex vivo experiments using peripheral blood mononuclear cells from patients treated with glofitamab demonstrate that T cells derived from responders exhibit significantly higher cytotoxic activity against lymphoma cells compared with those from non-responders. This increased cytotoxicity is accompanied by greater secretion of pro-inflammatory cytokines and cytotoxic granules, including interferon-γ, TNF-α, perforin, and granzyme B [56]. Consistent with these findings, responders also display greater induction of interferon-γ levels in peripheral blood after the first glofitamab administration, indicating a more robust systemic immune response. Interestingly, despite strong immune activation during therapy, longitudinal analyses suggest that repeated exposure to glofitamab does not irreversibly impair T-cell function. Peripheral T cells from responding patients maintain cytotoxic capacity across multiple treatment cycles, and preclinical studies demonstrate preserved or even enhanced effector activity of intratumoral T cells after repeated treatment. These findings suggest that durable responses may be sustained through ongoing recruitment of fresh T cells from the peripheral circulation rather than relying exclusively on pre-existing intratumoral clones.

### 3.3. T-Cell Exhaustion and Activation-Induced Dysfunction

Emerging evidence indicates that BsAbs may activate a broad polyclonal T-cell repertoire. Single-cell T-cell receptor sequencing analyses have shown that glofitamab treatment does not lead to the preferential expansion of dominant pre-existing clones in peripheral blood [56]. Instead, therapy appears to activate a diverse population of T cells, resulting in the expansion of multiple small and intermediate-sized clones rather than the emergence of dominant clonal populations. These findings support the concept that BsAbs engage the overall T-cell pool in a polyclonal manner, which may reduce dependence on pre-existing tumor-specific T cells [56].

Prior therapeutic exposure profoundly shapes systemic immune fitness, ultimately determining how effectively T cells respond to bispecific antibody (BsAbs) therapy. Patients with heavily pretreated large B-cell lymphoma (LBCL) frequently exhibit compromised T-cell fitness due to prior chemotherapy, lymphodepleting regimens, and chronic immune activation. Age, cumulative treatment exposure, and the interval since the last therapy can influence the abundance of naïve T cells and the degree of T-cell exhaustion [57]. In particular, shorter intervals between prior therapies and the initiation of BsAbs treatment have been associated with poorer T-cell fitness and inferior responses [57]. Finally, strategies aimed at enhancing T-cell fitness may represent promising approaches to overcome resistance. Preclinical studies have demonstrated that providing costimulatory signals through the 4-1BB pathway can increase the proportion of naïve-like and functionally competent intratumoral T cells during bispecific antibody therapy [58]. The combination of glofitamab with a CD19-targeted 4-1BB ligand agonist results in greater accumulation of functional T cells within the tumor microenvironment and improved antitumor activity compared with glofitamab alone [58].

Collectively, these findings suggest that T-cell exhaustion in the context of BsAb therapy reflects both a pre-existing determinant of response and a dynamic, therapy-induced state, rather than a purely causal or consequential phenomenon. Baseline differences in T-cell composition and fitness may predispose patients to suboptimal responses, while sustained CD3 engagement and chronic antigen stimulation during therapy further drive activation-induced dysfunction, progressively reinforcing exhausted phenotypes over time.

## 4. Microenvironmental Barrier

The immunosuppressive cellular landscape in DLBCL is composed of various specialized populations that facilitate immune evasion, promote tumor growth, and drive resistance to therapy. While the tumor microenvironment (TME) presents a multifaceted barrier, the PD-L1-mediated checkpoint axis and ATR-driven spatial exclusion within dark zone (DZ)-like niches represent the most critical determinants of therapeutic failure, as they respectively neutralize effector T cells and physically prevent the formation of the required immunological synapse. Within this hierarchy, macrophages are a dominant component of the DLBCL TME, particularly in the activated B-cell subtype [59]. M2-polarized macrophages are classically associated with immunosuppression and tumor progression, largely through the secretion of CCL18, which induces T-cell anergy and promotes a Th2-skewed, pro-tumorigenic milieu (Figure 2) [60].

### 4.1. Checkpoint Ligand Upregulation

The overexpression of PD-L1 within the DLBCL microenvironment serves as a critical mechanism of resistance that impairs the efficacy of TCEs by inducing T-cell dysfunction, promoting immune evasion, and acting as a physical and functional barrier to redirected T-cell activity. High levels of PD-L1 on the surface of DLBCL cells interact with PD-1 on tumor-infiltrating lymphocytes (TILs), leading to T-cell exhaustion characterized by reduced proliferation and impaired cytokine production [61]. The upregulation of PD-L1 is paradoxically triggered by the therapeutic action of TCEs and BsAbs. When these agents successfully bridge T cells to malignant cells, the resulting T-cell activation leads to the release of interferon-gamma (IFN-γ), which acts as a potent regulator that induces or increases PD-L1 expression on the surface of both malignant and healthy B cells [53,62]. This TCE-induced PD-L1 expression impairs the activity of PD-1-expressing T cells, effectively creating a feedback loop that shields the tumor from T-cell-mediated cytotoxicity and tumor cell lysis [53]. This upregulation is not limited to the surface of malignant cells; rather, it is a pervasive feature of the entire TME.

Beyond the PD-L1 axis, secondary co-inhibitory checkpoints such as CTLA-4 and TIM-3 further reinforce the TME’s inhibitory niche. CTLA-4 suppresses anti-tumor immunity by outcompeting CD28 for costimulatory ligands and physically removing CD80 via transendocytosis, a process that drives progressive T-cell exhaustion and correlates with resistance in R/R DLBCL [50,62,63,64]. Similarly, TIM-3 serves as a hallmark of terminal exhaustion and a baseline biomarker for non-response to epcoritamab. Notably, while PD-1^+^TIM-3^+^ CD8^+^ TILs within tumor clusters exhibit impaired proliferation and cytokine production (IFN-γ, TNF-α), they retain high levels of cytotoxic molecules. This suggests that these terminally exhausted populations, while currently a barrier to BsAb efficacy, remain functionally reactivable through therapeutic intervention [50,65,66,67,68].

### 4.2. Physical and Spatial Exclusion

The spatial and physical architecture of the TME in DLBCL creates an immunosuppressive landscape by co-opting the organizational logic of the lymphoid germinal center. A prominent feature of aggressive DLBCL is the retention of germinal center (GC) dark zone (DZ)-like characteristics, where a specialized immunological niche facilitates rapid B-cell proliferation and DNA diversification while actively excluding T cells [69,70]. This spatial exclusion is not necessarily dependent on physical barriers like the structured stroma found in epithelial cancers, but is instead driven by biochemical and epigenetic programs, specifically the activation of the ATR (ataxia telangiectasia and Rad3 related) kinase. High ATR activity in DZ-like regions orchestrates the DNA damage response (DDR) and promotes increased chromatin compaction, which creates a chromatin-mediated environment that limits T-cell infiltration [70]. Because BsAbs require direct tumor T-cell contact, this spatial exclusion may represent an important mechanism of therapeutic resistance.

While the traditional cell-of-origin (COO) classification (ABC/GCB) provides prognostic value for chemotherapy, it is insufficient to predict resistance to BsAbs [71,72]. A more precise framework involves the DLBCL Immune Quadrant (DLBCL-IQ), which segregates tumors into ABC hot, ABC cold, GCB hot, and GCB cold clusters based on their endogenous immune environment [73]. Integration of the LymphGen probabilistic classifier reveals that specific genetic subtypes orchestrate these “hot” or “cold” niches. GCB hot tumors, which are inherently susceptible to BsAb-mediated T-cell engagement, are significantly enriched for SOCS1 loss-of-function mutations that render B cells hypersensitive to IFN-γ, creating an inflamed microenvironment that is inherently susceptible to BsAb-mediated T-cell engagement [73]. Conversely, GCB cold tumors represent a major niche of resistance, often characterized by a dark zone signature and the EZB MYC+ genetic subtype, demonstrating extremely poor outcomes with mosunetuzumab, reaching a PFS of only 2.3 months [71]. Within the ABC subtype, ABC cold tumors are predominantly associated with the A53 (TP53/Aneuploidy) and MCD/BN2 (BCR drivers like MYD88 and CARD11) subtypes. These oncogenic pathways drive potent MYC activity, which facilitates the acquisition of a cold or T-cell-excluded microenvironment. In contrast, ABC hot tumors are enriched in the N1 (*NOTCH1*) subtype and frequently harbor CD274 (PD-L1) alterations, suggesting that while they attract T cells (immune-hot), they utilize checkpoint signaling to induce functional resistance [71].

Within these “immune-cold” regions, there is a lack of engaged immune checkpoints, with the notable exception of the PVRIG/NECTIN-2 axis, which may control the rare T cells that manage to infiltrate the DZ [74]. Spatial mapping further identifies a hierarchical topology where tumor cells form tight, layered clusters; the “core” of these clusters represents an immune desert entirely devoid of T-cell infiltration, while the “mantle” or “crust” regions are primarily dominated by macrophages [75]. In contrast, diffuse niches allow for better intermixing of tumor B cells and reactive T cells, but these areas are often associated with immune evasion mutations in MHC-I genes [69].

This physical and spatial compartmentalization poses a significant challenge to T-cell-based therapies, as these treatments require direct proximity to tumor cells to form functional immunological synapses. The intrinsic resistance of DZ-like DLBCL to these therapies is rooted in this spatial programming of immune exclusion, which prevents effector T cells from reaching the dense tumor core [70]. Even when T cells successfully navigate the TME, they often encounter immunosuppressive niches that lack essential recruitment signals such as CXCL9 and CXCL12, leading instead to high expression of markers for T-cell dysfunction and exhaustion like PD-1, TIGIT, and LAG-3 [69]. ATR inhibition (ATRi) may reverse this immune-silent state by disrupting chromatin compaction and flaring inflammatory signaling, thereby reprogramming the DZ-like TME into an immune-permissive environment that enhances T-cell recruitment and sensitizes tumor cells to killing [70]. Additionally, targeting the CXCR3 axis has been proposed as a strategy to force T-cell penetration into the restricted tumor core regions, potentially overcoming the spatial barriers that currently limit the success of cellular therapies in DLBCL [75]. Ultimately, the high selective pressure of TCE-mediated killing can drive the outgrowth of these spatially protected clones, highlighting the need for combination strategies that address both the biochemical and the structural barriers of the DLBCL microenvironment.

### 4.3. Hypoxia and Metabolic Suppression

Hypoxia and metabolic suppression within the DLBCL TME are increasingly recognized as emerging mechanisms of resistance to novel immunotherapies, although their precise contributions remain incompletely understood. The inherently hypoxic nature of lymphoid tissues, further exacerbated by rapid tumor growth, leads to vascular insufficiency, focal necrosis, and stabilization of HIF-1α, driving adaptive metabolic reprogramming in malignant cells. Under these conditions, DLBCL cells appear to suppress global protein synthesis while selectively upregulating glycolytic mediators such as GLUT1 and hexokinase 2 (HK2), thereby sustaining aerobic glycolysis and intensifying competition for key nutrients like glucose and glutamine, which may limit T-cell effector functions, including IFN-γ and granzyme B production [76,77]. Concurrently, lactate accumulation and extracellular acidification are proposed to impair T-cell activation by disrupting NFAT signaling, while also promoting the immunosuppressive polarization of macrophages and stabilization of FoxP3 in regulatory T cells (Tregs), potentially reinforcing local immune suppression. Hypoxia has also been associated with increased PD-L1 expression on tumor and myeloid cells, suggesting a link to enhanced PD-1-mediated inhibitory signaling and immune escape [44]. Collectively, these interconnected metabolic and hypoxic adaptations represent a complex and evolving area of investigation, warranting further study to clarify their therapeutic implications.

## 5. Strategies to Overcome Resistance to Bispecific Antibodies

Despite encouraging clinical activity, a substantial proportion of DLBCL patients treated with CD20 × CD3 BsAbs experience primary resistance or relapse after an initial response. Resistance conceivably arises from complex interactions between tumor-intrinsic mechanisms, T-cell dysfunction, and the immunosuppressive features of the TME. Consequently, multiple strategies are being explored to overcome these barriers and enhance the durability of responses to BsAbs [78].

### 5.1. Targeting Tumor-Intrinsic Resistance Mechanisms

Effective activity of CD20 × CD3 BsAbs depends on sustained expression of the target antigen on malignant B cells. Loss or downregulation of CD20 expression has been increasingly recognized as an important mechanism of resistance following BsAbs therapy [26].

Therapeutic strategies aimed at restoring antigen expression are therefore being actively investigated. Epigenetic modulation represents one promising approach. Histone deacetylase inhibitors and EZH2 inhibitors have been shown to increase CD20 expression and enhance the susceptibility of lymphoma cells to BsAbs-mediated killing [79]. Preclinical studies demonstrate that epigenetic priming can simultaneously increase CD20 surface expression and activate T-cell responses, thereby improving tumor cell sensitivity to CD20 × CD3 antibodies [79,80,81]. Another strategy to mitigate antigen escape involves targeting multiple tumor antigens simultaneously. The development of multispecific antibodies or trispecific constructs capable of engaging two tumor antigens together with CD3 may reduce the likelihood of antigen loss-mediated resistance. Such approaches are currently under investigation and aim to provide more robust tumor targeting in a variety of lymphomas, including DLBCL [82].

### 5.2. Restoring T-Cell Function and Reversing Exhaustion

T-cell fitness is a critical determinant of response to BsAbs therapy. Persistent antigen exposure and continuous CD3 engagement during treatment can drive T-cell exhaustion, characterized by impaired proliferative capacity, reduced cytokine production, and sustained expression of inhibitory receptors such as PD-1, LAG-3, and TIM-3. Therapeutic strategies that reinvigorate exhausted T cells are therefore being explored as a means of improving BsAbs efficacy [50]. One approach involves combining TCEs with immune checkpoint inhibitors targeting inhibitory pathways such as PD-1 or CTLA-4 [83,84,85,86,87]. Preclinical studies and early clinical trials suggest that checkpoint blockade can restore T-cell activation and enhance tumor cell killing when used in combination with BsAbs [88]. Cytokine support represents another promising strategy. Cytokines such as interleukin-2 and interleukin-15 can promote T-cell survival, proliferation, and effector function, potentially enhancing the persistence of BsAbs-activated T cells [50]. These cytokines may help sustain T-cell responses and counteract exhaustion during prolonged therapy. More recently, innovative approaches have been proposed to directly target exhaustion pathways. For example, nanoparticle-based bispecific TCEs capable of delivering an adenosine A2A receptor antagonist have been developed to counteract the immunosuppressive adenosine signaling pathway [89]. In preclinical models, this strategy reduced T-cell exhaustion and significantly improved tumor cell killing compared with conventional BsAbs [89].

### 5.3. Targeting the Tumor Microenvironment

The tumor microenvironment plays a critical role in modulating the efficacy of T-cell-redirecting therapies. Strategies aimed at modulating the TME are therefore being actively investigated [50]. Depletion of regulatory T cells has been shown in experimental models to enhance the efficacy of TCEs and convert nonresponders to responders [50]. Similarly, targeting suppressive myeloid populations or metabolic pathways, such as the adenosine signaling axis, may improve immune activation within the tumor microenvironment [90]. Tumor microenvironmental features, such as stromal signaling, hypoxia, and immunosuppressive cytokines, have also been associated with resistance to TCEs. These findings suggest that combination therapies aimed at reversing microenvironmental immunosuppression may enhance the efficacy of BsAbs therapy.

### 5.4. Rational Combination Therapies

Combination therapy represents one of the most promising strategies to enhance the clinical activity of BsAbs in DLBCL [78]. Because BsAbs can be administered repeatedly and have manageable toxicity profiles, they can be readily combined with other therapeutic modalities. Several classes of agents are currently being investigated in combination with CD20 × CD3 BsAbs. Immunomodulatory drugs such as lenalidomide may enhance T-cell activation and cytokine production while promoting immune synapse formation [91,92]. Bruton tyrosine kinase inhibitors may further augment T-cell function and improve immune signaling pathways. In addition, antibody–drug conjugates, such as polatuzumab vedotin, can increase tumor antigen release and enhance immune activation. These combinations aim to create a more favorable immune contexture that enhances T-cell-mediated tumor killing [93,94]. BsAbs are also being explored in combination with other targeted agents (Table 1) [42,78,95,96,97]. These combinations may increase tumor antigen exposure, enhance immune infiltration, and reduce tumor burden, thereby improving the efficacy of T-cell redirection.

Overall, these strategies target complementary mechanisms of resistance; however, the currently available evidence remains limited and largely derived from preclinical or early-phase studies and does not yet allow a clear hierarchical prioritization of the different approaches.

### 5.5. Next-Generation BsAbs Designs

Advances in antibody engineering are also driving the development of next-generation T-cell-redirecting therapies designed to overcome resistance mechanisms [98]. Novel bispecific formats with optimized structural properties may improve immune synapse formation, enhance T-cell activation, and reduce treatment-related toxicity. Trispecific antibodies incorporating additional costimulatory signals represent one such approach [16,99]. By providing both T-cell receptor engagement and costimulatory signaling, these molecules may promote sustained T-cell activation while limiting exhaustion. Additionally, novel delivery platforms and antibody designs that integrate immune-modulatory payloads may further enhance antitumor immune responses.

**Table 1:** Ongoing clinical trials evaluating BsAbs in combination with targeted agents in DLBCL.

Trial Name	Phase	Population	Experimental Arm	Study Design	Required CD20+	NCT
CLEAR	II	R/R DLBCL	Epcoritamab + Loncastuximab teserine	Single arm	No	NCT07197307
ECLAT	II	R/R DLBCL	Epcoritamab + Lenalidomide + Tafasitamab	Single arm	Yes	NCT07030699
REPIFIR	II	R/R DLBCL	Epcooritamab + Lenalidomide + Tafasitamab	Single arm	No	NCT07126236
EPCORE DLBCL-4	III	R/R DLBCL	Epcoritamab + Lenalidomide	Multi arm	No	NCT06508658
EPCORE DLBCL-3	II	Newly diagnosed DLBCL	Epcoritamab + Lenalidomide	Two arm	Yes	NCT05660967
	I/II	R/R DLBCL	Epcoritamab + Lenalidomide or Epcoritamab + Lenalidomide + Ibrutinib	Two arm	Yes	NCT05283720
	II	R/R DLBCL	Glofitamab + Chidamide	Single arm	No	NCT06570447
	II	Newly diagnosed DLBCL	Glofitamab + Polatuzumab vedotin + Zanubrutinib +	Single arm	Yes	NCT07012980
	II	Newly diagnosed DLBCL	Glofitamab + Axicabtagen Ciloleucel or Relmacabtagene Autoleucel	Multi arm	Yes	NCT07326371
	II	Newly diagnosed DLBCL	Glofitamab + Polatuzumab Vedotin + Zuberitamab	Single arm	Yes	NCT07231250
	II	R/R DLBCL	Glofitamab + Lenalidomide + Radiotherapy	Single arm	Yes	NCT06651853
	II	Newly diagnosed DLBCL	Glofitamab + Polatuzumab vedotin + Zanubrutinib + Lenalidomide	Single arm	No	NCT06665217
	II	R/R DLBCL	Mosunetuzumab + CAR-T	Single arm	No	NCT04889716
	I/II	Newly diagnosed DLBCL	Mosunetuzumab + polatuzumab vedotin	Single arm	Yes	NCT03677154
	II	R/R DLBCL	Mosunetuzumab + Loncastuximab Vedotin	Single arm	Yes	NCT05672251
	I	Richter transformation	Odronextamab + Zanubrutinib	Single arm	Yes	NCT06735664

## 6. Conclusions

Bispecific antibodies (BsAbs) have emerged as an important therapeutic option for patients with relapsed or refractory DLBCL, providing an off-the-shelf approach to redirect endogenous T cells against malignant B cells. Despite encouraging clinical activity, both primary and acquired resistance remain major challenges that limit the durability of response. Similar to CAR T-cell therapy, resistance reflects overlapping mechanisms of immune escape, including antigen modulation, T-cell dysfunction, and an immunosuppressive tumor microenvironment; however, BsAbs are uniquely dependent on the baseline quantity, spatial distribution, and functional competence of the endogenous T-cell compartment, rather than on the expansion and persistence of an engineered cellular product. Available evidence supports a conceptual—albeit still evolving—hierarchical model of resistance to CD20 × CD3 BsAbs. Tumor-intrinsic factors such as CD20 loss or modulation represent an important layer, but are insufficient to fully explain resistance, as many patients relapse despite preserved antigen expression. This observation highlights the central role of T-cell fitness and functionality, including exhaustion, impaired cytotoxicity, and skewing toward immunosuppressive subsets, as key determinants of therapeutic failure. At a higher level, the tumor microenvironment acts as a dominant regulator by shaping T-cell infiltration, spatial organization, and checkpoint-mediated suppression, thereby conditioning the effectiveness of T-cell redirection. Although this framework remains limited by the heterogeneity and relative scarcity of translational data, it provides a useful model for integrating currently known resistance mechanisms. A deeper understanding of these interconnected layers will be critical to refine patient selection and guide therapeutic strategies. Future progress will likely rely on composite biomarker approaches integrating antigen expression, T-cell phenotypic and functional profiles, and microenvironmental features, as well as on rational combination therapies and next-generation bispecific constructs designed to enhance T-cell function, overcome immune suppression, and remodel the lymphoma microenvironment.

## Data Availability

No new data were created or analyzed in this study. Data sharing is not applicable to this article.
